# Can Knee Arthroscopy Be Considered Entirely Safe for Patients Over 50 Years Old With no Risk of Osteonecrosis? Case Series and Literature Review on Post‐Artrhoscopy Osteonecrosis of the Knee (PAONK)

**DOI:** 10.1111/os.70020

**Published:** 2025-03-17

**Authors:** Panagiotis Ntagiopoulos, Georgios Kalinterakis, Pierrenzo Pozzi, Dimitris Fligkos, George Themistocleous, Sotirios Themistokleous, Triantafyllia Dimou, Riccardo Compagnoni, Paolo Ferrua, Pietro Simone Randelli

**Affiliations:** ^1^ Hip and Knee Unit, Mediterraneo Hospital Athens Greece; ^2^ U.O.C. 1° Clinica Ortopedica, ASST G. Pini‐CTO Milan Italy; ^3^ Department of Biomedical, Surgical and Dental Sciences Università Degli Studi di Milano Milan Italy; ^4^ Department of Biomedical Sciences for Health Università Degli Studi di Milano Milan Italy

**Keywords:** arthroscopy, chondral lesions, knee, meniscectomy, osteonecrosis

## Abstract

**Objective:**

Although post‐arthroscopy osteonecrosis of the knee is well‐documented in the literature, its etiology and prognosis remain unclear. The purpose of this study is to present a group of individuals who experienced avascular necrosis following knee arthroscopy, to examine the factors leading to this condition and assess the outcomes of treatment, as well as to perform a literature review on the subject.

**Methods:**

We retrospectively studied patients between January 2015 and March 2024 who had developed knee osteonecrosis following a standard arthroscopic procedure for treating meniscal tears. All adult patients with isolated meniscus tears and grade 2 or less chondral lesions were included. Patients with evidence of bone edema on MRI performed 4–6 weeks after the onset of preoperative symptoms were not included in the study. The Knee injury and Osteoarthritis Outcome Score (KOOS) was used as an outcome measure. A correlation analysis was performed to explore the degree of association between variables, with significance set at *p* < 0.05.

**Results:**

Eight patients out of 974 arthroscopies met the inclusion criteria. There was one woman and seven men (mean age 57 [range: 51–71]). The lesions noted at arthroscopy included seven medial meniscus tears that were treated with excision and one lateral meniscal tear that was treated with suture repair and still developed osteonecrosis. None of them were traumatic while all patients had early degenerative changes in the compartment of interest.

**Conclusions:**

Osteonecrosis should be suspected in older patients experiencing worsening symptoms following knee arthroscopy for degenerative meniscus tears and partial meniscectomy. Increased age, a higher BMI, and a delayed diagnosis appear to be associated with more severe disease progression and the need for operative treatment.

## Graphical abstract

Post‐arthroscopy osteonecrosis is a rare complication occurring in 0.82% of cases, primarily affecting patients over 50 with degenerative meniscal tears. Early detection through MRI and conservative treatment, including bisphosphonates and weight‐bearing avoidance, proved effective in most cases, while some required surgical intervention. The findings underscore the importance of vigilance in older patients undergoing knee arthroscopy.

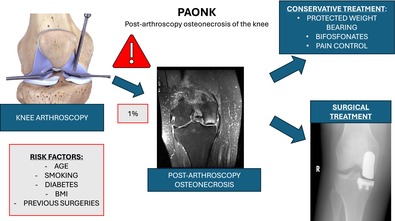

## 1 Introduction

Spontaneous osteonecrosis of the knee is a condition marked by the lack of blood flow leading to the death of bone marrow and the trabecular layer of the subchondral bone [1]. The development of osteonecrosis after arthroscopic knee surgery is a rare yet serious complication. Brahme was the first to describe it in 1991, recognizing it as a possible complication of arthroscopic meniscectomy [2]. Since then, a larger population has been described with the terms “post‐arthroscopic” and “post‐meniscectomy” osteonecrosis (PAO). To establish the diagnosis of post‐arthroscopy osteonecrosis, there must be no bone marrow edema on a preoperative magnetic resonance imaging (MRI) 4–6 weeks prior to surgery and must be present on postoperative MRI [3]. Although there is a correlation between meniscectomy and the development of bone marrow edema leading to osteonecrosis, this condition has also been observed following other arthroscopic procedures, including ACL reconstruction and chondroplasty [4, 5, 6]. Furthermore, osteonecrosis has frequently been observed following laser‐assisted or radiofrequency‐assisted arthroscopic debridement procedures [7, 8, 9]. It remains uncertain whether meniscectomy alone can lead to osteonecrosis or if the combination of mechanical and radiofrequency chondroplasty plays a role in its development. Because of the low incidence of the disease, the exact aetiopathogenesis of this condition is not certain. In the literature, there are only a few case series regarding this entity, most of which do not evaluate the results of osteonecrosis treatment. Some authors either do not routinely perform preoperative MRIs or they perform them outside the “window frame”: in this way, the existence of preoperative osteonecrosis may have been missed, and it might be impossible to differentiate between PAO and early spontaneous osteonecrosis of the knee (SPONK). The purpose of this study is to (i) report the incidence of post‐surgical osteonecrosis in patients who underwent arthroscopic meniscal surgery, (ii) identify potential underlying factors, and (iii) document the outcomes of its treatment.

## 2 Methods

This retrospective study was conducted in line with the Declaration of Helsinki, and the protocol received approval from the Ethics Committee of Mediterraneo Hospital (33/15‐05‐2023). Informed consent was obtained from all patients for their participation in the study. From January 2015 to June 2023, a total of 974 arthroscopic meniscectomies were performed at our clinic. Preoperative and postoperative clinical notes, plain films, and magnetic resonance images of the patients were meticulously reviewed. The study's inclusion criteria were as follows:

1. Skeletally mature patients,
2. Isolated meniscus tears treated with arthroscopy, with MRI findings indicating a torn meniscus, such as increased signal within the body of the meniscus that communicates with the superior and/or inferior surface,
3. Chondral lesions of grade 2 or less,
4. No evidence of preoperative bone edema on MRI performed 6 weeks or more after the beginning of the symptoms.

Exclusion criteria included patients with meniscal lesions and combined ligamentous injuries with bone bruises or advanced arthritic lesions.

Post‐arthroscopy osteonecrosis was diagnosed based on the presence of bone marrow edema (BME) on a postoperative MRI, which was absent on a preoperative MRI performed 4–6 weeks after the start of symptoms. If the preoperative MRI was conducted within the first 6 weeks, the possibility of underlying spontaneous osteonecrosis of the knee (SPONK) cannot be excluded. Additionally, it is crucial to differentiate this condition from transient bone marrow edema, a common MRI finding, particularly after a meniscectomy, which typically has a benign outcome.

The MRI criteria that suggest early irreversible osteonecrosis rather than transient lesions include:

1. Subchondral area with low signal intensity on T2‐weighted images,
2. Focal depression of the epiphyseal contour,
3. Low signal intensity lines are located deep within the affected condyle [10].

The patients were analyzed with respect to age, gender, BMI, associated diseases, symptoms at presentation, previous surgeries, and factors that increased their vulnerability to the disease such as smoking, alcohol, and corticosteroid use. A corticosteroid dosage exceeding 2 g per month for at least 3 months was identified as a risk factor [11]. For alcohol consumption, more than 400 mg per week was considered a possible hazard [12]. Regarding tobacco consumption, smoking more than 10 cigarettes per day for a 10‐year period was associated with an increased risk of developing the disease [13]. The clinical data were collected prospectively by the author (G.K.) from the time of presentation and diagnosis of osteonecrosis of the knee. All images were typically assessed by one expert radiologist. All the surgeries were performed by the senior author (P.G.N.) under spinal anesthesia. A tourniquet at 300 mmHg was applied in all patients. For meniscectomies, standard arthroscopic instruments were used (e.g., baskets forceps and 4.5 mm shaver). Full weight‐bearing was immediately allowed after surgery, and a standard rehabilitation protocol was followed, including quadriceps strengthening, proprioception exercises, ice therapy, and progressive return to daily activities and sports after 6 weeks. Follow‐up visits were scheduled at 3 weeks, 1.5 months, and 12 months after the operation.

Clinical evaluation for patients with osteonecrosis was conducted using the Knee Injury and Osteoarthritis Outcome Score (KOOS). Symptomatic post‐arthroscopy patients were assessed with anteroposterior and lateral standing radiographs, as well as MRI, to determine the size and location of the osteonecrotic lesion. The size of the osteonecrotic lesion was measured on T1‐weighted images using Lotke's method, where the area of low signal intensity was expressed as a percentage of the diameter of the medial femoral condyle [14]. Long‐standing anteroposterior radiographs were performed to evaluate axial alignment. All symptomatic patients with post‐arthroscopy bone edema were treated conservatively. Initial treatment consisted of avoiding weight bearing for 1.5 to 3 months, using painkillers (paracetamol) and taking bisphosphonates (alendronate sodium and cholecalciferol 70 mg/2800 IU weekly). At 6 and 12 weeks, a new clinical evaluation was performed, and a new MRI was conducted. In cases of symptom deterioration and progression to osteonecrosis, surgical treatment was followed according to patients age and mechanical axis (e.g., unicompartmental knee arthroplasty, high tibial osteotomy); specific surgical treatment is beyond the scope of the present study.

## 3 Literature Review

An extensive literature review of all published articles was conducted to correlate our results with the previously published case series. The search was performed from inception to May 31, 2024. Two databases were used: PubMed and Scopus. We considered for inclusion case reports, case series, and retrospective and prospective clinical trials related to osteonecrosis of the knee following arthroscopic conventional meniscectomy for meniscal tears. Animal and cadaveric studies as well as non‐English studies were excluded. The search keywords used were arthroscopy, osteonecrosis, avascular necrosis, knee, meniscectomy, meniscal tears, post‐arthroscopy, and post‐meniscectomy. Each paper was independently reviewed by two authors (G.K. and D.F.).

Descriptive statistics are presented for numerical demographic data, and the categorical variables are reported as percentages. A point‐biserial correlation analysis was used to evaluate the relationship between the risk factors and the outcome. We specifically examined how the following variables—age, BMI, smoking, corticosteroid use, alcohol consumption, delay in osteonecrosis diagnosis, size of the osteonecrotic lesion, and the number of knee areas with chondral damage—correlated with whether the patient received conservative or operative treatment. A *p* < 0.05 was considered statistically significant. Data were collected and analyzed using Microsoft Excel and SPSS v27 (IBM Corp., Armonk, NY).

## 4 Results

### 4.1 Pre‐Operative Patient Data Analysis

Eight patients out of 974 arthroscopies met the inclusion criteria (prevalence of 0.82%). There was one woman and seven men. Their average age was 57 years (range: 51–71 years). The mean BMI was 26.3 kg/m^2^ (range: 21.4–29 kg/m^2^). The preoperative symptoms included persisting for more than 6 weeks joint line pain, nocturnal pain, compartment pain, and intermittent effusion in the knee. As far as medical history is concerned, neither of these patients had stated any trauma history before the start of symptoms. None of the patients showed signs of osteoarthritis on plain radiographs. Two patients exhibited varus malalignment of the knee. Additionally, no signs of osteonecrosis were present on the initial radiographs. Three out of eight patients (37%) were heavy smokers for a 10‐year period (more than 10 cigarettes per day) while none mentioned corticosteroid use or alcohol consumption. All patients were initially treated non‐operatively with physiotherapy and painkillers, and alendronic acid for a minimum of 6 weeks. An MRI was ordered in case of no improvement with conservative treatment. A retrospective analysis of this subgroup of patients with post‐arthroscopy osteonecrosis revealed that the period of their symptoms before undergoing knee MRI ranged from 1.5 to 6 months. The extent of conservative treatment before knee arthroscopy varied from 1 to 10 months. The interval between the MRI and the knee arthroscopy procedure ranged from 1.5 to 6.5 weeks, with an average of 5.5 weeks. Pre‐arthroscopy MRI confirmed isolated meniscal pathology in all eight cases. It revealed a medial meniscus tear in seven knees (87.5%) and a lateral meniscus tear in one knee (12.5%) before the arthroscopic procedure. Chondral damage (outerbridge grade I/II) was identified in five knees. Importantly, no signs of osteonecrosis were observed on any pre‐arthroscopy MRI.

### 4.2 Intraoperative Findings

In these patients, operative findings included medial meniscus tears in seven cases and a lateral meniscus tear in one knee. Outerbridge grade I/II chondromalacia of the patella was observed in six knees (75%), grade II of the medial tibial condyle in four knees (50%), grade II of the medial femoral condyle in seven knees (87.5%) and grade II of the lateral femoral condyle in one knee (12.5%). Abrasion chondroplasty or microfractures were avoided, and instead, chondral lesions were not addressed surgically. Partial meniscectomy was performed for all meniscal tears except one, which was treated with all‐inside sutures. Radiofrequency was not used in any of these cases. Tourniquet use was standardized in all patients. The average tourniquet time was 13.5 min (range: 10–17 min). An arthroscopic pump was used in all knees.

### 4.3 Post‐Operative Clinical and Imaging Analysis

**FIGURE 1:**
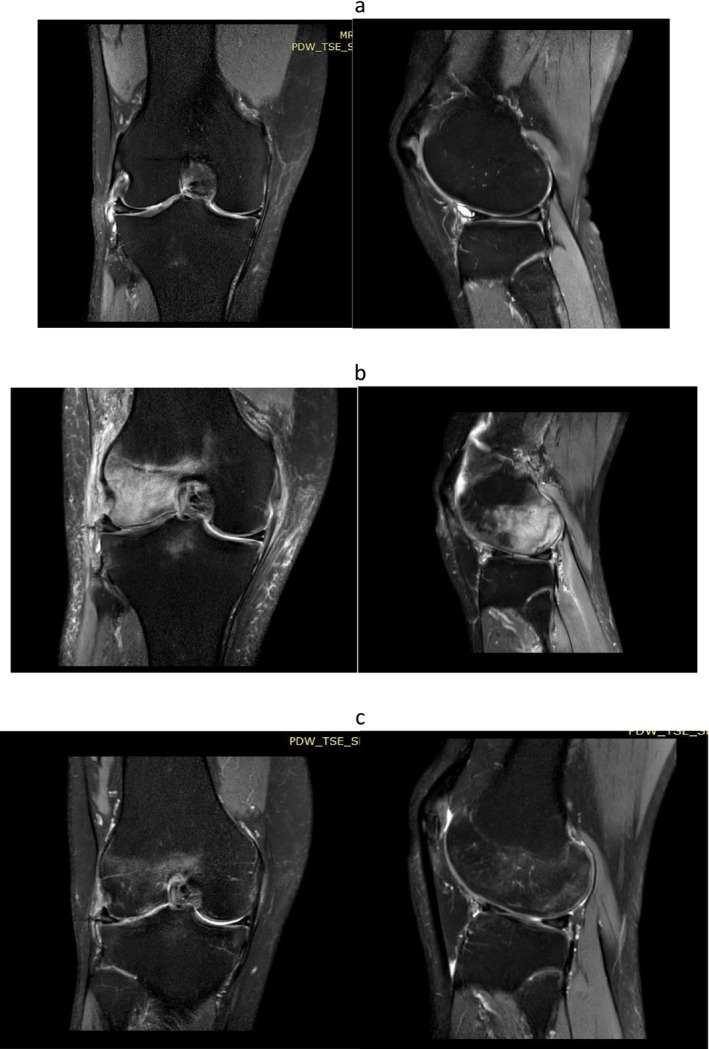
(a) Pre‐operative T2‐weighted images depicting the lateral meniscus tear and normal bone, (b) Post‐operative T2‐weighted images 3 weeks after the surgery, demonstrating the abnormal signal in the lateral femoral condyle, (c) Post‐operative T2‐weighted images demonstrating the subsidence of the lesion at the final follow‐up.

**FIGURE 2:**
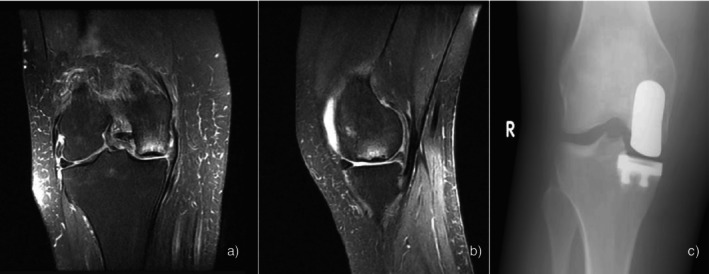
(a) Coronal and (b) sagittal T2‐weighted magnetic resonance images showing a focal subchondral patch of increased bone marrow signal of the medial femoral condyle. (c) Anteroposterior radiograph showing the knee after UNI.

The same pattern of symptom onset was observed after arthroscopy; all patients reported that despite initial relief, their symptoms worsened as their activity levels increased after 4 weeks. All eight patients underwent an MRI to assess their ongoing pain, with the MRI performed an average of 3.2 months after surgery (range: 1.5–6 months). The MRI revealed osteonecrosis of the medial femoral condyle in seven patients, while in one patient, osteonecrosis involved the lateral femoral condyle (Figure 1). Regarding the tibial plateau, it was involved only in two patients. Findings of osteonecrosis were evident on plain radiographs in three patients, mostly presenting as subchondral lucency. Notably, the location of post‐arthroscopy osteonecrosis corresponded in the compartment of meniscal pathology in all patients. After the diagnosis, all eight patients were treated with protected weight bearing, painkillers, and bisphosphonates. Patients were administered alendronate 70 mg weekly for 3–4 months. Three out of eight patients failed to respond to conservative treatment and underwent unicompartmental arthroplasty (UNI) which eventually resolved their symptoms (Figure 2). Four patients responded to conservative treatment after an average of 12 weeks of non‐weight, partial weight bearing, and bisphosphonates administration (range: 5–24 weeks). In one patient, no signs of osteonecrosis improvement were present on MRI; nonetheless, symptoms resolved, and no further action was required at the 3‐year follow‐up. All patients were monitored for 1 year. The KOOS scores are provided in Table 1.

**TABLE 1:** Epidemiological and clinical data of reviewed cases.

Age	Sex	BMI (kg/m^2^)	Mechanical axial deviation	Duration of symptoms prior to MRI	Findings on MRI	Findings on arthroscopy	Procedure done/tourniquet time	Post‐op MRI	Time between arthroscopy and post‐op MRI	Subsequent treatment	KOOS before the 1st operation	KOOS after diagnosis of ON	Post‐KOOS/last follow up
71	M	28.1	Medial	6 months	CL‐GI MFC, CL‐GI MTP, CL‐GII patella, MMT	CL‐GII MFC, CL‐GI MTP, CL‐GII patella, MMT	PMM/10 min	ON‐MFC/MTP	2.5 months	UNI	52	40	82
58	F	25.8	Neutral	2 months	CL‐G I MFC, CL‐GI patella, MMT	CL‐G II MFC, CL‐GI patella, MMT	PMM/15 min	ON‐MFC	1 month	Conservative	49	39	78
63	M	27.4	Neutral	4.5 months	CL‐G II MFC, CL‐GI patella, MMT	CL‐G II MFC, CL‐GII patella, MMT	PMM/12 min	ON‐MFC	3 weeks	Conservative	58	44	83
68	M	28.7	Medial	1.5 months	CL‐GII MFC, CL‐GI MTP, CL‐GI patella, MMT	CL‐GII MFC, CL‐GI MTP, CL‐GI patella, MMT	PMM/12 min	ON‐MFC	1.5 month	UNI	62	52	84
62	F	29	Neutral	2 months	CL‐GII MFC, CL‐GI MTP, CL‐GI patella, MMT	CL‐GII MFC, CL‐GII MTP, CL‐GII patella, MMT	PMM/15 min	ON‐MFC/MTP	2 months	UNI	52	45	80
55	M	26.2	Neutral	4 months	CL‐GII MFC, MMT	CL‐GII MFC, MMT	PMM/12 min	ON‐MFC	1 month	Conservative	50	42	79
54	M	21.4	Neutral	3 months	CL‐G II LFC, CL‐GI patella, LMT	CL‐G II LFC, CL‐GII patella, LMT	MR/17 min	ON‐LFC	3 weeks	Conservative	49	40	84
51	M	24.1	Neutral	6 months	CL‐G II MFC, CL‐GII MTP, MMT	CL‐G II MFC, CL‐GII MTP, MMT	PMM/15 min	ON‐MFC	1 month	Conservative	54	48	87

Abbreviations: CL‐G, chondral lesion‐grade; LFC, lateral femoral condyle; LMT, lateral meniscus tear; LTP, lateral tibial plateau; MFC, medial femoral condyle; MMT, medial meniscus tear; MR, meniscal repair; MTP, medial tibial plateau; ON, osteonecrosis; PMM, partial medial meniscectomy; UNI, unicompartmental knee arthroplasty.

### 4.4 Correlation Analysis

The correlations between the examined variables are shown in Table 2. Based on the analysis results, there was a positive, strong, and significant correlation between the variables: age, BMI, and delay in diagnosis with the outcome (conservative versus operative treatment). Consequently, older patients with a higher BMI and delayed diagnosis of osteonecrosis were at a higher risk for operative treatment. In contrast, smoking, size, and number of the lesions were not significantly correlated with the outcome.

**TABLE 2:** Pearson correlations between six risk factors and outcome.

Variable	1	2	3	4	5	6	7
1. Age	—						
2. BMI	0.77*	—					
3. Smoking	0.24	0.45	—				
4. Delay of diagnosis	0.71*	0.65	0.46	—			
5. Size of lesion	0.45	0.86*	0.32	0.56	—		
6. Number of chondral lesions	0.28	0.45	0.91*	0.67	0.45	—	
7. Outcome	0.79*	0.72*	0.20	0.89*	0.66	0.46	—

* Correlation is significant at the 0.05 level (two‐tailed).

### 4.5 Literature Review

Regarding the literature review, there were a total of 14 studies, 8 of which were case series, and the rest were case reports [2, 15, 16, 17, 18, 19, 20, 21, 22, 23, 24, 25, 26, 27]. The total patient number was 61. Men outnumbered women, with 55.8% and 44.2%, respectively. The mean age of the patients was 60.3 (range: 34–81). MRI was used as the initial diagnostic tool in all studies. The majority of patients (90.3%) had a medial meniscal tear identified in the pre‐operative MRI. In 6 out of 14 studies, the exact onset of clinical symptoms before the preoperative MRI was not reported, while in three studies, the preoperative MRI was conducted during the “window period.” As a result, around 31 out of 62 patients may have had an undiagnosed early‐stage spontaneous osteonecrosis of the knee prior to the procedure. The area affected consistently matched the location of the arthroscopic surgery. In all cases, the lesion size was less than 25%. Osteonecrosis development was predominant in the femoral condyle (91.9%) compared to the tibial plateau (8.1%). As far as the final treatment is concerned, 30 cases were treated operatively, as opposed to 23 whose symptoms subsided with conservative measures. Regarding the type of surgical treatment, 20 patients underwent arthroplasty (TKA/UNI), 8 patients were treated with revision arthroscopy, and 2 patients underwent high tibial osteotomy, while one study did not provide specific information about the treatment.

## 5 Discussion

### 5.1 Incidence

Post arthroscopic osteonecrosis is a rare yet serious (in respect to the initial cause of admission) complication of knee arthroscopy, and it has been particularly reported in patients undergoing meniscectomy. Given the large number of arthroscopic meniscectomies performed globally each year, the prevalence of post‐arthroscopy osteonecrosis appears to be quite low. Our study confirms previous studies estimating incidence between 0.2% and 1.5% (0.82%) [28].

### 5.2 Diagnosis

Pre‐operative evaluation with an MRI is a prerequisite for the diagnosis of post‐arthroscopy osteonecrosis to be established. Based on the study of Nakamura et al., there is a consensus that the minimum length between the beginning of the symptoms and the MRI examination, used as an inclusion criterion, was set at 6 weeks [29]. Otherwise, the differential diagnosis between spontaneous and post‐arthroscopic osteonecrosis is difficult to be done. Interestingly, over half of the reviewed studies (9 out of 14) either failed to specify the exact onset of symptoms before the MRI or conducted the MRI during the “window period.” As a result, the accuracy of the diagnosis was considered uncertain. However, all the cases included in our study met this criterion.

### 5.3 Risk Factors

This condition shows a preference for elderly patients diagnosed with degenerative medial meniscus tears. It is of note that the majority of them had pre‐arthritic knees. The medial femoral condyle was predominantly affected. Furthermore, in all studies, the location of osteonecrosis was geographically correlated with the pre‐existing pathology and the sites of arthroscopic procedures. As mentioned before, it is essential for this entity to be differentiated from spontaneous osteonecrosis. These two conditions appear to have different risk factors. Spontaneous onset osteonecrosis is a condition of females older than 65 years [30, 31]. Regarding post‐arthroscopic osteonecrosis, men are affected more commonly than women. The size of the lesion as a percentage of the diameter of the medial femoral condyle is a significant factor in predicting outcomes and guiding treatment for spontaneous osteonecrosis, but this correlation is rarely seen in post‐arthroscopic osteonecrosis [28]. In our study, the average size of the lesion was less than 30% for all patients, possibly due to the short time period between the initial arthroscopy and the MRI diagnosis of post‐arthroscopic osteonecrosis (3.2 months). The latter is in accordance with the literature results. As for the age, both conditions affect mainly the elderly. However, post‐arthroscopic osteonecrosis has a slightly wider age distribution ranging from 34 to 81 years (Table 3).

**TABLE 3:** Published cases of post‐arthroscopic osteonecrosis of the knee associated with meniscectomy.

Author	Type of study	Number of patients	Gender (M/F)	Mean age (range)	Meniscal tears (M/L)	Mean duration between symptoms and diagnostic MRI before initial arthroscopy	Primary operation	Number of patients with chondromalacia at initial arthroscopy	Number of patients where chondromalacia treated with chondroplasty	ON location involved	Treatment (conservative/operative)
Brahme et al. [2]	Case report	7	4/3	60.5 (42–72)	6/1	N/C	6PMM/1PLM	7MFC/1LFC/1MTP	7	6MFC/1MTP/1LFC	2/5
Santorini et al. [15]	Case series	2	1/1	34 (21–47)	2/0	N/C	2PMM	_	0	2MFC	2/0
AI‐Kaar et al. [16]	Case series	10	5/5	69 (55–81)	9/1	N/C	9PMM/1PLM	4MFC/1LFC	7	9MFC/1LFC	7/3
Prues‐Latour et al. [17]	Case series	9	4/5	69 (58–82)	8/1	6.5 months	8PMM/1PLM	4MFC/2LFC 3MTP/1LTP	7	8MFC/1LFC 1MTP	6/3
Johnson et al. [18]	Case series	7	3/4	60 (41–79)	4/3	10.5 months (1.5–36)	4PMM/2PLM	7MFC/2LFC 3MTP/4LTP	6	4MFC/1MTP/1LFC/1LTP	1/5
Faletti et al. [19]	Case report	1	1/0	66	1/0	N/C	PMM	_	0	MFC	0/1
Kushayama et al. [20]	Case series	2	2/0	52	2/0	2.5 weeks in one case	PMM	_	0	2MFC	2/0
DeFalco et al. [21]	Case report	1	1/0	48	1/0	3 weeks	PMM	_	0	MFC	0/1
Musculo et al. [22]	Case series	8	3/5	65 (54–75)	8/0	N/C	5PMM/3TMM	4MFC	4	8MFC	N/A
Akgunetal. [23]	Case series	4	2/2	57.5 (34–78)	4/0	2 months in 2 cases	PMM	1MFC	2	4MFC	3/1
MacDessi et al. [24]	Case series	7	5/2	64 (53–78)	8/0	N/C	PMM	1MFC/1MTP	3	7MFC/1MTP	0/8
Son et al. [25]	Case report	1	1/0	50	1/0	5 days	PMM	MFC	0	MFC	0/1
Zhuang et al. [26]	Case report	1	1/0	81	1/0	3 months	PMM	_	0	MFC	0/1
Alomar [27]	Case report	1	1/0	69	1/0	6 months	PMM	_	0	MFC	0/1
Total	14 studies	61	34/27	60.3 (mean)	56/6				36		23/30

Abbreviations: LFC, lateral femoral condyle; LMT, lateral meniscus tear; LTP, lateral tibial plateau; MFC, medial femoral condyle; MMT, medial meniscus tear; MTP, medial tibial plateau; N/C, not clear; ON, osteonecrosis; PMM, partial medial meniscectomy.

In our study as opposed to those reviewed, we also examined possible risk factors that are associated with secondary osteonecrosis of the knee. Three of the eight patients had known risk factors for secondary osteonecrosis, specifically a history of smoking. Nonetheless, even if there was a positive correlation between smoking and surgical treatment, the relationship was not significant. Other risk factors such as corticosteroid and alcohol use have not been present. In contrast to post‐arthroscopic osteonecrosis, secondary osteonecrosis usually affects younger patients, especially women less than 45 years of age. It is located more commonly in the hip joint and may be bilateral in up to 80% of the cases. The pain is presented gradually, and it is rarely managed non‐operatively [32].

### 5.4 Etiopathogenesis

So far, the medical literature has not provided a clear explanation for the cause of osteonecrosis that occurs following arthroscopic surgery. The most plausible hypothesis, which is also supported by the authors of this article, is that after meniscectomy there is a change in the way the weight is distributed across the knee joint. This change would lead to higher pressure at the tibiofemoral contact point, causing small fractures and tears in the cartilage, which then could allow synovial fluid to leak into the subchondral bone. If there is pre‐existing arthritis in that specific area of the joint, this process would be accelerated due to the reduced ability of the cartilage to absorb impact [33, 34]. In the present study, one patient developed osteonecrosis after meniscal repair with sutures. As far as we are concerned, this is the first case described in the literature confirming our concerns about the complexity of etiopathogenesis. However, this finding is preliminary, and additional research is required to validate the observation. Other causes include iatrogenic chondral damage and the use of laser or radiofrequency during arthroscopy [35].

### 5.5 Treatment

In terms of treatment, our literature research (Table 3) revealed that over half of the patients (56.6%) required surgical intervention. However, in our study, most patients were successfully treated non‐operatively with satisfied KOOS scores at the last follow‐up. It is worth noting that the size of the lesion did not seem to impact the disease's progression, as all lesions in our patients were small in size. Conversely, patients who underwent surgery tended to have a delayed diagnosis compared to those who did not. It appears that if osteonecrosis is diagnosed early, a favorable outcome can be achieved with conservative treatment. In addition to the latter, age and BMI might have been predictors of treatment success or failure, as there was a strong positive correlation between these variables and the surgical treatment. Therefore, the surgeon must have a low threshold for obtaining a new post‐arthroscopy MRI, especially in older and overweight patients with persisting knee symptoms for over 4 weeks after meniscectomy.

At present, there is no universally agreed‐upon or evidence‐based set of guidelines for treatment. However, a commonly followed approach is to initiate non‐operative treatment as soon as the diagnosis is confirmed, consisting of an extended period of non‐weight bearing and the use of standard pain relievers. Research has indicated that bisphosphonates can be advantageous in the non‐surgical treatment of post‐arthroscopic osteonecrosis by halting the resorption process in the necrotic area [36, 37, 38]. In our study, all patients were commenced on bisphosphonates, so we believe that they may play a role in preventing surgery, either by bone edema resorption or by stopping progression to osteonecrosis. Surgical intervention should be contemplated if patients do not show improvement clinically or radiographically after 3–4 months of non‐operative treatment [39, 40]. A recent nationwide study on surgical trends in managing knee osteonecrosis revealed that total knee arthroplasty (TKA) was the most frequently performed procedure, followed by UNI [41]. In our case, three patients underwent unicompartmental arthroplasty that resolved symptoms.

### 5.6 Strengths and Limitations

The major advantage of this study is that all patients were assessed pre‐operatively with MRI, which was conducted 6 weeks after the onset of symptoms. So, the possibility of an underlying osteonecrosis has been eliminated. Another strength is the evaluation of risk factors and clinical outcomes. However, this study is not without limitations. First and foremost, its retrospective design makes finding a comparison group extremely difficult. Second, as the data was collected from medical records not originally intended for research purposes, some important information may be missing. Thus, while this study suggests an association between post‐arthroscopic osteonecrosis and degenerative torn meniscus in elderly patients with early underlying arthritis in the affected compartment, it is important to note that a direct causal relationship may not be definitively established. The development of post‐arthroscopic osteonecrosis is likely influenced by a combination of complex and multifactorial factors. Furthermore, MRI images were reviewed by one radiologist, so the inter‐reader reliability was not assessed, introducing possible systematic errors and distorting conclusions. Finally, no blinding was implemented in terms of clinical evaluations, radiological assessments, and data analysis. This may have introduced reporting and detection biases, making the results less reliable and the conclusions not fully generalizable. Additional prospective studies are required to define the relevant patient risk factors leading to this entity and to evaluate the treatment.

## 6 Conclusions

Currently, there are no established evidence‐based guidelines that enable arthroscopic surgeons to accurately predict or prevent the occurrence of true post‐arthroscopic knee osteonecrosis following partial meniscectomies. However, given the rising number of aging athletes undergoing arthroscopy, it is crucial for surgeons to maintain a high level of awareness of this rare complication, particularly in elderly patients with meniscal tears and pre‐existing knee arthritis. Increased age, higher BMI, and delayed diagnosis appear to be associated with more severe disease progression and the need for operative treatment. Adequate pre‐operative patient education should also be emphasized in these cases.

## Author Contributions

**Panagiotis Ntagiopoulos:** conceptualization, methodology, supervision, validation, writing – review and editing. **Georgios Kalinterakis:** conceptualization, data curation, formal analysis, investigation, methodology, writing – original draft, writing – review and editing. **Pierrenzo Pozzi:** formal analysis, validation, writing – original draft, writing – review and editing. **Dimitris Fligkos:** investigation, validation, writing – review and editing. **George Themistocleous:** investigation, supervision, validation, writing – review and editing. **Sotirios Themistokleous:** investigation, methodology, writing – review and editing. **Triantafyllia Dimou:** validation, visualization. **Paolo Ferrua:** methodology, validation, writing – review and editing. **Riccardo Compagnoni:** validation, visualization, writing – review and editing. **Pietro Simone Randelli:** supervision, writing – review and editing.

## Ethics Statement

This study was approved by the ethical committee of Mediterraneo Hospital, Athens, Greece.

## Consent

All patients and their parents were informed and consented to their participation in the study.

## Conflicts of Interest

The authors declare no conflicts of interest.
